# Enhanced On-Demand Antibacterial Platform Based on Triboelectric-Nanogenerator-Induced Electrical Stimulation of Cu_2_S Substrates

**DOI:** 10.1021/acsami.5c04717

**Published:** 2025-07-21

**Authors:** Marziyeh Jannesari, Leyla Shooshtari, Nima Mohamadbeigi, Niall J. English, Raheleh Mohammadpour

**Affiliations:** ‡ School of Chemical and Bioprocess Engineering, 8797 University College Dublin, Belfield, Dublin D04 V1W8, Ireland; § Center for Nanoscience and Nanotechnology, Institute for Convergence Science and Technology, 68260 Sharif University of Technology, 14588- 89694 Tehran, Iran; ⊥ Semiconductor Department, Materials and Energy Research Center, 31787-316 Karaj, Iran

**Keywords:** triboelectric nanogenerators (TENGs), self-power, on-demand antibacterial, electron transport chain (ETC), reactive oxygen species (ROS), Cu_2_S, Cu_2_O

## Abstract

Triboelectric nanogenerators (TENGs) offer a sustainable, battery-free solution for wearable electronics by converting motion into energy. However, direct skin contact poses bacterial contamination risks, requiring advanced antibacterial strategies. This study developed an on-demand antibacterial platform based on TENG-induced electrical stimulation of Cu_2_S substrates, benchmarked against Cu_2_O. Submicron-structured Cu_2_S layers were fabricated via a novel sulfurization method applied to electrodeposited Cu_2_O layers on fluorine-doped tin oxide (FTO) substrates. The resulting Cu_2_S and Cu_2_O thin films were integrated into a single-electrode TENG system, and their antibacterial efficacy was evaluated under electrical stimulation driven by a Kapton–FTO TENG.Experimental results revealed that a 10-min finger-tapping-generated electrical current from the TENG significantly enhanced the antibacterial performance of Cu_2_S, increasing its efficacy against bacterial models of *Staphylococcus aureus* and *Escherichia coli* from 25% to 70% and from 55% to 100%, respectively. In contrast, Cu_2_O demonstrated high intrinsic antibacterial activity with minimal improvement under TENG stimulation. The enhanced response of Cu_2_S was attributed to a ∼115% increase in Cu ion release, significantly higher than the ∼17% increase observed for Cu_2_O. This enhanced performance was further attributed to intensified electrostatic interactions between positively charged electrode surfaces and negatively charged bacterial membranes, leading to membrane interruption and bacterial death. Additionally, electron capture from bacterial electron transport chains heightened oxidative stress, disrupted energy metabolism, and further enhanced antibacterial effects. These findings accentuate the potential for integrating TENGs into biomedical applications, particularly in advanced wearable devices, to provide inherent antibacterial functionality for safe and effective direct human contact.

## Graphical abstract

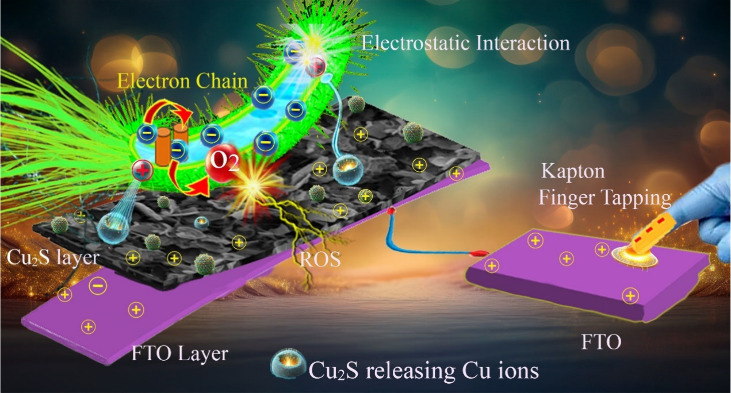

## 1 Introduction

The World Health Organization (WHO) has identified antimicrobial resistance (AMR) as one of humanity’s Top Ten global public health threats.^1^ The misuse and overuse of antibiotics in various fields such as human medicine, agriculture, and animal husbandry have augmented the emergence of bacterial-resistant strains, rendering conventional antibiotics infective.^2,3^ The rising prevalence of antibiotic-resistant bacteria has sparked significant interest in exploring alternative antimicrobial approaches. This concerning trend has led to increased burdens, both in terms of higher mortality rates and escalating healthcare costs. In light of these challenges, there is an urgent need for innovative, sustainable, and targeted antimicrobial strategies that minimize unnecessary exposure to antibacterial agents. On-demand antibacterial systems, which activate only under specific conditions, represent a promising solution. By enabling spatially and temporally controlled antibacterial action approaches,^4^ these systems are supporting global efforts to reduce the risk of resistance development associated with continuous or excessive use of antimicrobial agents.^5^

Physical strategies like electrical stimulation, which involve applying electrical currents, have been introduced to control and combat pathogenic bacteria.^6^ These approaches with physical treatment aim to provide controllable on-demand antibacterial properties. More recently, triboelectric nanogenerators (TENGs), known for their exceptional ability to harvest electrical energy from mechanical motion and friction, generating electric stimulation have emerged as an up-and-coming technology for antibacterial activities.^7,8^ TENGs not only provide numerous advantages such as customized design, consistent operation, and cost-effectiveness but also feature self-power capabilities. This eliminates the need for external energy sources, thereby enhancing their practicality and sustainability.^9,10^ Furthermore, TENG flexibility facilitates effortless integration with the body’s movements, making them ideal for adaptable wearable applications.^11^ TENGs can also be incorporated into a variety of surfaces and materials, supporting their use in medical devices, wound dressings,^12^ and everyday items designed to minimize bacterial contamination.^13^ Importantly, endowing these devices with antibacterial properties is critical, as recent studies have reported that electronic and wearable devices pose a substantial risk of bacterial colonization, particularly by skin-associated pathogenic bacteria. For example, pathogenic microorganisms were identified on nearly 26% of personal electronic devices in clinical settings^14^ and more recently, metagenomic sequencing was used to reveal widespread contamination, including multidrug-resistant strains, on wearable electronics used by healthcare workers.^15^ These findings highlight the urgent need for on-demand antibacterial solutions in wearable technology to mitigate the risk of microbial transmission and ensure safer use in healthcare and daily life applications.

Recent studies have emphasized that the effectiveness of TENG-based electrical stimulation devices in combating bacterial infections is intricately linked to the chemical properties of the bacteria-hosting surface.^16^ Several strategies have been reported to impart antibacterial properties to TENG devices. The primary approach involves using materials with specific chemical structures that possess inherent antibacterial properties. Chitosan, a natural polymer,^17^ and polyaniline nanofibrous films^18^ have demonstrated significant bactericidal effects when used in TENGs due to its inherent antibacterial properties. Polypyrrole, a conductive polymer that is widely used as a TENG electrode, can also provide antibacterial activity by its nature. Its positive charges enable the polymer to interact electrostatically with the negatively charged surfaces of bacteria, effectively fighting them.^19^ Bromobutyl rubber also has been reported as an inherent antibacterial active in TENG devices.^17^ Another strategy involves incorporating antibacterial materials, including silver (Ag),^20,21^ copper (Cu),^22^ zinc oxide (ZnO),^23^ and titanium dioxide (TiO_2_) nanoparticles,^24^ into the electrode substrate. These nanoparticles enhance the antibacterial efficacy of TENG devices by leveraging their unique properties to disrupt bacterial cells and inhibit their growth.

Although these strategies can provide antibacterial treatment using materials with inherent antibacterial properties, it is advantageous to have antibacterial properties activated at specific times only when needed. This on-demand targeted approach helps reduce the risk of inducing bacterial resistance associated with the excessive or continuous presence of antibacterial agents. By enabling antibacterial activity only when needed, TENG-based electrical stimulation offers a compelling strategy to minimize the overuse of such agents. This advancement positions TENGs as a promising platform for controlled and responsive antibacterial treatments.

By utilizing TENGs, localized electric fields generated through mechanical activation, disrupt the integrity of bacterial cell membranes, leading to the leakage of cellular contents and eventual cell death.^25^ Furthermore, reactive oxygen species (ROS) generated by TENGs can induce oxidative stress in bacteria, thereby enhancing their antibacterial effect.^7,26^ This targeted activation mechanism confines antibacterial effects to specific infection sites, minimizing harm to healthy tissues. Moreover, such systems optimize the delivery of antibacterial agents, thereby enhancing treatment efficacy.^27^ Beyond clinical benefits, integrating TENG-based electrical stimulation into medical devices and wound dressings showcases its potential to revolutionize infection management, offering sustainable and environmentally friendly alternatives to conventional antibiotic therapies. This highlights the transformative impact of on-demand antibacterial treatments on improving patient outcomes across diverse healthcare settings.

Despite recent advancements in TENG-based antibacterial technologies, a significant gap remains in the development of on-demand antibacterial systems that synergistically combine electrical stimulation with functional host materials whose antibacterial properties are selectively activated or enhanced under electric fields. In particular, limited attention has been given to how the chemical modification or conversion of functional groups in the host electrode materials can modulate their interaction with bacteria under electrical stimulation. This unexplored area presents a unique opportunity to tailor antibacterial responses by controlling the electrochemical behavior of the electrode, thus achieving precise, electrically triggered bactericidal effects without continuous material-based toxicity.

Bearing in mind these open questions regarding antibacterial mechanisms, this study is driven by the objective of chemically modifying Cu_2_O electrodes through a novel sulfurization strategy to form Cu_2_S, and to investigate how this structural transformation influences the on-demand antibacterial properties activated by electrical stimulation specifically, the electric field generated by an integrated triboelectric nanogenerator system.

Cu_2_S possesses excellent semiconducting properties, making it particularly well-suited for integration into energy-harvesting systems such as triboelectric nanogenerators, where charge transfer efficiency and surface interactions are critical. In addition to its functional performance, various studies have consistently demonstrated that Cu_2_S exhibits an acceptable level of biocompatibility, both in vitro^28^ and in vivo,^29,30^ when in contact with a range of mammalian cell types. Notably, this includes studies involving fibroblast (NIH/3T3) cells,^30^ which are widely used as a representative model for skin tissue in biocompatibility assessments. The favorable biocompatibility results, combined with the relatively low cytotoxicity of Cu_2_S compared to other heavy-metal-based materials, suggest that it can be safely applied in wearable TENG devices designed for direct skin contact. Moreover, the cost-effectiveness of Cu_2_S fabrication further enhances its potential for use in scalable, skin-contact wearable technologies, positioning it as a promising candidate for the development of skin-friendly, long-term wearable electronics. The Cu_2_S layer was synthesized through the sulfurization of a thin Cu_2_O layer, already electrodeposited on a fluorine-doped tin oxide (FTO) substrate. While significant advancements have been made in sulfurization techniques for copper-based materials, the transformation of Cu_2_O to Cu_2_S via sulfurization on FTO substrates remains underexplored. Most previous studies have focused on sulfurization methods involving polysulfide electrolytes, such as aqueous solutions containing precise concentrations of Na_2_S and elemental sulfur.^31^ This work presented a novel sulfurization method to fabricate Cu_2_S layers from electrodeposited Cu_2_O on FTO. This innovative approach not only provides valuable insights into the synthesis of Cu_2_S but also highlights its significant bioapplication potential, particularly in antibacterial treatments requiring rapid and efficient response.

In this study, we aim to develop on-demand antibacterial functionality in Cu-based substrates using an integrated triboelectric nanogenerator (TENG) system. The setup employed a Kapton-wrapped finger as a single-electrode TENG (S-TENG), generating electric current upon tapping the FTO substrate. This current was transferred to either Cu_2_O or Cu_2_S substrates (hosting bacteria) deposited on the FTO. The FTO layer served as an electrode, ensuring electrical connectivity within the system. In this study, FTO was deliberately selected as the control substrate due to its chemical stability, biological inertness, lack of significant intrinsic antibacterial activity, and, importantly, its cost-effectiveness and scalability. These characteristics make FTO an ideal baseline material, enabling a clearer interpretation of the antibacterial effects arising specifically from the Cu-based layers and/or the TENG-induced electrical stimulation, without interference from the substrate itself. The TENG-based antibacterial properties of the Cu_2_O and Cu_2_S substrates were evaluated comprehensively using two bacterial models of *Escherichia coli* and *Staphylococcus aureus*, representing Gram-negative and Gram-positive bacteria, respectively. This approach demonstrated the superior on-demand antibacterial efficacy of Cu_2_S under TENG-based electrical stimulation compared to its precursor, electrodeposited Cu_2_O, as a bacterial host material, highlighting the exceptional potential of Cu_2_S in antibacterial applications, in response to electrical stimuli. It is worth mentioning that the bacterial models are standard laboratory strains that are not specifically antibiotic-resistant. However, the antibacterial mechanism investigated is independent of antibiotic susceptibility, suggesting that the platform may have potential applicability against antibiotic-resistant strains in future studies.

## 2 Results and Discussion

### 2.1 Material Characterization

The crystal structure of the synthesis materials was investigated using X-ray diffraction (XRD) pattern analysis with a PANalytical X’Pert PRO instrument in the range of 20–70°. XRD analysis of Cu_2_O shown in Figure 1A was conducted to investigate the crystal phase of Cu_2_O nanoparticles. The diffraction peaks at 2θ = 29.6°, 36.4°, 42.3°, 52.5°, and 61.3° correspond to the (110), (111), (200), (211), and (220) planes, respectively. These peaks match well with the standard reference for copper­(I) oxide (JCPDS 05-0667), confirming the cubic structure of Cu_2_O. The intensity and sharpness of the peaks indicate high crystallinity, while the absence of impurity peaks suggests phase purity. This characterization confirms the well-defined structural properties of Cu_2_O nanoparticles.

**Figure 1:**
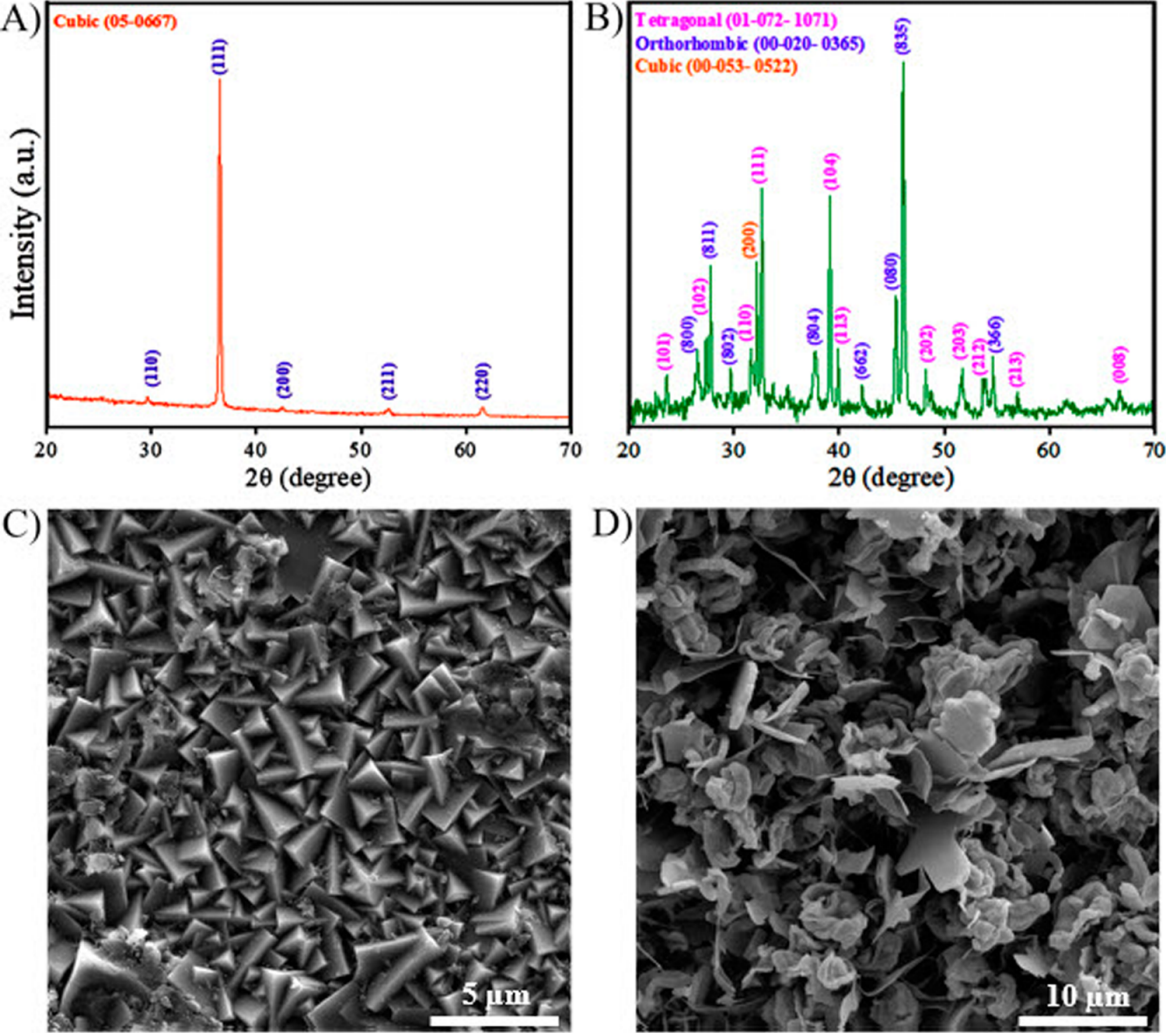
XRD patterns of the (A) Cu_2_O and (B) Cu_2_S layers. FESEM images of the (C) Cu_2_O and (D) Cu_2_S layers. The Cu_2_S layer was synthesized by sulfurizing the Cu_2_O layer, which was grown on an FTO substrate through an electrodeposition process.

The XRD analysis of Cu_2_S is shown in Figure 1B; the characteristic diffraction peaks at 2θ values of 23.6°, 27.3°, 31.6°, 32.6°, 39.1°, 39.9°, 45.4°, 46.0°, 48.2°, and 51.7° were well indexed to the Cu_2_S structure, matching the standard card JCPDS 01-072-1071. These peaks correspond to the (101), (102), (110), (101), (104), (113), (200), (201), (202), and (203) planes, respectively, with calculated lattice parameters of *a* = 3.99 Å and *c* = 11.28 Å, consistent with a tetragonal structure. Additionally, peaks at 26.6°, 29.6°, 31.6°, and 37.9° matched the Cu_2_S structure according to JCPDS 00-002-1294, corresponding to the (341), (090), (282), and (139) planes, respectively, with lattice parameters *a* = 11.8 Å, *b* = 27.2 Å, and *c* = 22.7 Å, indicating an orthorhombic structure. Further diffraction peaks at 27.7°, 32.1°, 46.0°, 51.7°, and 54.6° were indexed to the Cu_2_S structure in accordance with JCPDS 00-053-0522, corresponding to the (111), (200), (220), (310), and (331) planes, respectively, with a cubic structure and a lattice parameter of *a* = 5.56 Å. Additionally, based on the Debye–Scherrer equation, which is *D* = *K*λ/β cos θ,^32^ where *D* is the nanoparticle crystalline size, *K* represents the Scherrer constant (0.98), λ denotes the wavelength, and β denotes the full-width at half-maximum (fwhm). The crystallite size was estimated to use the Debye–Scherrer equation, yielding an average size of 42.6 nm. These findings confirm the high purity and crystallinity of Cu_2_S, highlighting its potential for applications in photovoltaics and thermoelectric devices.

The morphological characteristics of Cu_2_O and Cu_2_S were investigated using field-emission scanning electron microscopy (FESEM). The FESEM image of Cu_2_O (Figure 1C) reveals a distinct pyramidal morphology, consistent with previous reports.^33^ Interestingly, upon exposure to the sulfurizing process at 500 °C, the Cu_2_O structure transforms into a Cu_2_S structure (Figure 1D), reflecting a significant morphological change induced by the sulfurization process. FESEM analysis of the Cu_2_S layer (Figures 1D and S1) supports the XRD findings, revealing a heterogeneous morphology characterized by layered and petal-like structures interspersed with compact regions. The orthorhombic phase corresponds to the anisotropic, layered features, while the tetragonal phase is likely responsible for the petal-like structures, and the cubic phase contributes to the uniform compact areas. Furthermore, regions of particle agglomeration were observed, likely due to the high surface energy inherent to nanoscale particles.

### 2.2 TENG Output Studies

In this study, the TENG employs a single-electrode, contact–separation design in which a Kapton film wrapped around a finger is repeatedly tapped against a FTO/glass substrate. This interaction generates surface charges due to the triboelectric effect, meaning that negative charges accumulate on the Kapton film, leaving behind positive charges on the FTO layer, in accordance with their positions in the triboelectric series. This charge exchange mechanism drives electrons through the external circuit, generating an alternating current. As shown in Figure 2A, the grounded FTO substrate serves as the TENG electrode and supports the Cu_2_O or Cu_2_S host layers, onto which bacteria are incubated for assessment of the system’s antibacterial performance. During the separation of the Kapton film and FTO layers, electrons are driven from the ground to the FTO substrate to balance the charge difference created by the triboelectric effect. At the maximum separation distance, the current ceases, indicating a pause in charge transfer. When the Kapton-wrapped finger moves closer to the FTO/glass surface, electrons flow back from the FTO electrode to the ground in the reverse direction, re-establishing charge equilibrium. This continuous back-and-forth motion of the Kapton film over the FTO/glass substrate induces cyclic variations in current flow within the system, an assurance of the TENG’s operational mechanism. The current and voltage amplitudes of the proposed TENG, generated by tapping the Kapton-wrapped finger on the FTO substrate at a constant frequency of ∼4 Hz (resulting in pulses recur approximately every 0.25 s), are presented in Figures 2B­(I),B­(II), respectively. A single voltage pulse generated by this TENG setup is shown in Figure S2. This frequency of ∼4 Hz was selected because it approximates a comfortable and repeatable manual tapping rate, aligning with typical actuation patterns in wearable or hand-held device applications. This makes the analysis relevant and potentially translatable to real-world use cases involving human interaction. The induced positive charges on the Cu_2_O or Cu_2_S layers are integral to the antibacterial mechanism, as these charges interact with the bacterial samples deposited on the host electrodes, facilitating electrical stimulation and antibacterial action.

**Figure 2:**
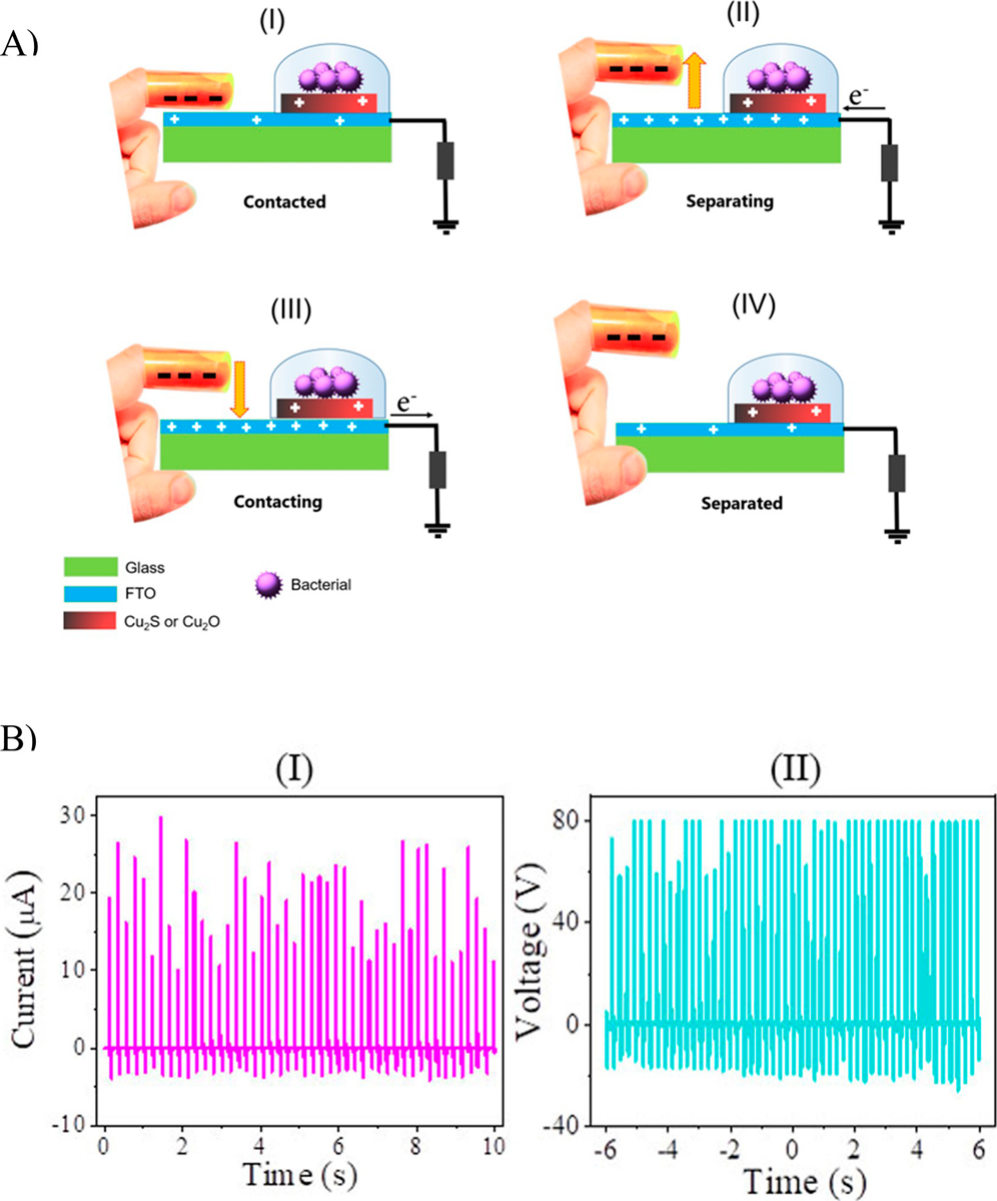
(A) Schematic illustration of the integrated single-electrode TENG, inducing an electrical effect on the bacteria incubated on either Cu_2_O or Cu_2_S via tapping of the finger-wrapped Kapton on the FTO (with a frequency of ∼4 Hz). (B) Evolution of the (I) current amplitude and (II) voltage during the time of the proposed TENG, showing pulses recurring every 0.25 s.

### 2.3 Antibacterial Studies

The antibacterial efficacy of the host substrates (Cu_2_O and Cu_2_S) induced by TENG-based electrical stimulation was investigated and compared with those not exposed to such stimulation. These investigations were conducted during the mid log phase of bacterial growth, characterized by a significant increase in population due to active cell division. This phase provided an ample population size, allowing for a robust evaluation of the effectiveness of antibacterial interventions. To monitor the response of bacterial models to the electrical stimulation, finger-tapping-based TENG was applied for 10 min and then, optical density (OD_600_) measurement and colony-forming-unit (CFU) counting methods were employed to investigate the antibacterial properties of the treatments. Furthermore, to validate the bactericidal properties of the treatments, a live/dead-staining assay was also conducted on both treated and untreated bacteria.

As demonstrated in Figure 3, the FTO substrate exhibited no bactericidal activity in the absence or presence of TENG-based electrical stimulation. This suggests that the substrate itself lacks inherent antibacterial properties. In contrast, FTO coated with Cu_2_O demonstrated potent intrinsic antibacterial effects, (particularly against Gram-negative bacteria *E. coli*), regardless of the presence of electrical stimulation, with further enhancement observed upon exposure to finger-tapped electrical effects, resulting in a 100% antibacterial effect. On the other hand, albeit another copper coating, Cu_2_S showed around 25 and 50% antibacterial activity by its nature, and the impact of electrical stimulation was more evident by inhibiting bacterial growth to 70 and 100% after receiving the TENG-based electrical effects on *S. aureus* and *E. coli* bacterial cells, respectively. This raises the question of whether the observed bactericidal activity of the copper-based electrodes is due to their intrinsic properties or if the electric effects induced by the finger-tapping TENG are playing a significant role.

**Figure 3:**
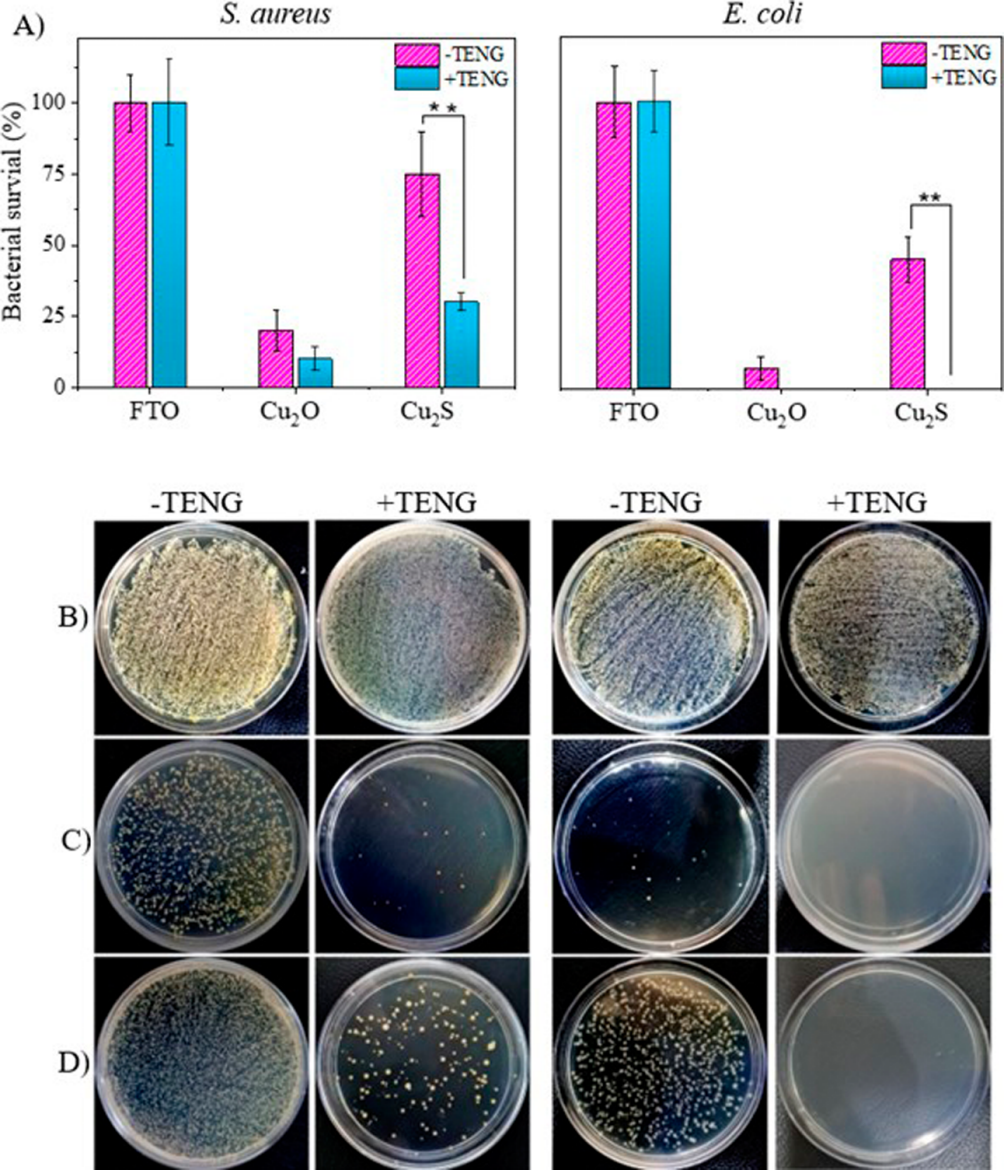
Antibacterial effects of different electrode hosts through (A) an optical density method and plate count agar on different substrates of (B) FTO, (C) Cu_2_O, and (D) Cu_2_S targeting bacterial models of *E. coli* and *S. aureus* under both nonstimulated conditions and with 10 min of TENG-based electrical stimulation. All antimicrobial experiments were independently performed in triplicate (*n* = 3), and the results are presented as mean ± SD. Asterisks (**) indicate statistically significant differences at *p* < 0.01.

To address this fundamental question, the bacteria, experiencing 10 min TENG-based electrical stimulation, remained in contact with the host electrodes in an agar medium overnight (with no further electrical stimulation). The findings illustrated in Figure 4 indicate that bacterial growth was completely inhibited in the presence of Cu_2_O electrodes, regardless of whether the electrodes underwent 10 min of TENG-based electrical stimulation. However, the situation is markedly distinct for Cu_2_S electrodes. In this case, the application of electric effects resulted in significant bactericidal activities of the Cu_2_S electrodes hosting the bacteria as compared to the electrodes, receiving no electrical stimulation. These findings reconfirm the significant enhancement of antibacterial properties in Cu_2_S electrodes only due to the electric stimulation induced by finger tapping.

**Figure 4:**
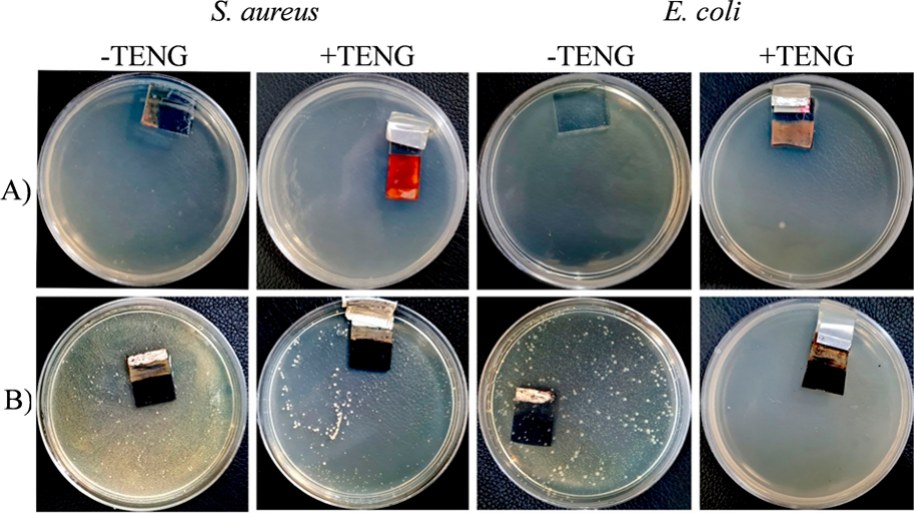
Overnight incubation of the bacterial models on the host electrodes of (A) Cu_2_O and (B) Cu_2_S with and without exposure to 10 min of TENG-based electrical stimulation.

Moreover, it was found that Gram-negative bacteria were more susceptible to both types of electrodes as well as the TENG-based electric effects. This susceptibility can be attributed to the physical and chemical nature of bacterial cells. Typically, Gram-positive bacteria such as *S. aureus* have thicker cell wall membranes containing golden carotenoid pigments.^34^ These structures can provide greater integrity and promote resistance against oxidative stress compared to Gram-negative bacteria whose cell membranes are thinner.^35,36^

### 2.4 Live/Dead Bacterial Staining Study

To further validate the observed antibacterial properties of the host electrodes with and without the finger-tap-induced TENG effect, fluorescent molecular probes were used to differentiate between live and dead *E. coli* bacterial cells. This technique allowed for a clear distinction between active and deactivated bacteria, providing insights into the effectiveness of the treatment. In this method, a dual probe staining approach was employed, utilizing propidium iodide (PI) and acridine orange (AO). Where PI selectively penetrates cells with compromised membranes, enabling the identification of damaged cells,^37^ AO can permeate both intact and damaged membranes, providing a broader assessment of cellular states.^38^ Upon staining, PI emits red fluorescence by interacting with DNA in bacterial cells with disrupted membranes, highlighting damaged cells. In contrast, AO stains live cells green, facilitating the clear visualization of intact, viable cells.

As illustrated in Figure 5, the intensified green emissions observed from bacteria, incubated on the FTO surface, in both the absence and presence of the finger-tapping TENG-based electrical stimulation, indicate that FTO does not effectively inhibit bacterial growth, even when subjected to electrical treatment.

**Figure 5:**
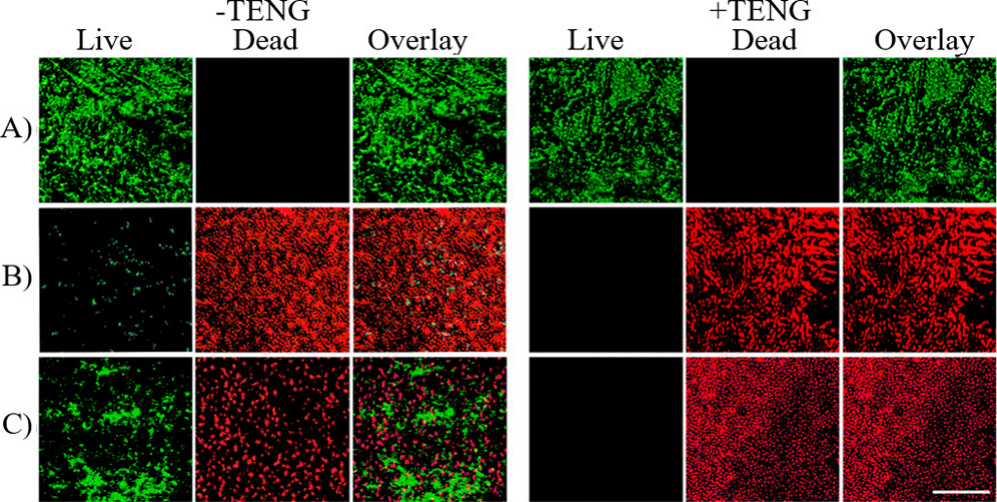
Fluorescent staining images of live/dead *E. coli* bacterial cells following exposure to (A) FTO, (B) Cu_2_O, and (C) Cu_2_S host substrates, both with and without TENG-based electrical stimulation for 10 min. The scale bar shows 100 μm.

For Cu_2_O host electrodes, the strong red fluorescence emitted by bacteria indicates the material’s inherent antibacterial properties. The increase in red fluorescence, accompanied by a notable reduction in green fluorescence, underscores the enhanced antibacterial efficacy achieved through TENG-based electrical stimulation in Cu_2_O electrodes. This effect was particularly pronounced when *E. coli* Gram-negative bacteria were exposed to Cu_2_S electrodes. The significant reduction in green fluorescence, coupled with a marked increase in red emission from the bacteria, highlights the superior bactericidal activity of Cu_2_S electrodes under TENG-based electrical stimulation.

For Cu_2_O host electrodes, the strong red fluorescence emitted from bacteria reflects the material’s inherent antibacterial properties. An increase in red emission, accompanied by a simultaneous depletion in green emission, highlights the enhanced antibacterial effect of TENG-based electrical stimulation in Cu_2_O electrodes. This effect was especially pronounced when *E. coli* Gram-negative bacteria were exposed to Cu_2_S electrodes. The observed decrease in green emission, along with a substantial increase in red fluorescence from the incubated bacteria, indicates a significant enhancement in the bactericidal activity of Cu_2_S electrodes under TENG-based electrical stimulation.

### 2.5 Morphological Investigations of the Bacteria by FESEM Analyses

FESEM was utilized to envision the changes in morphological structures of the bacteria, subjected to induced TENG-based electrical stimulation upon contact with the host electrodes. As depicted in Figure 6, the structures of both bacterial models in contact with FTO remained intact, regardless of receiving finger-tapping-based electrical stimulation treatment. Their membranes exhibited smooth and distinct boundaries, with no significant changes observed after exposure to the electrical stimulation. Upon exposure to Cu_2_O substrates, bacterial morphology underwent notable changes, with cells appearing blurred and rough-surfaced, showing clear signs of rupture and perforation. These structural alterations ultimately led to membrane collapse and the release of the extracellular matrix, emphasizing the bactericidal activity of Cu_2_O, especially when subjected to electrical stimulation. Similarly, FESEM images of bacteria on Cu_2_S electrodes revealed compromised membrane integrity, with surface irregularities, ruptures, and perforations alongside extracellular matrix release. These morphological disruptions were further intensified following TENG-based electrical stimulation treatment, reconfirming the superior antibacterial effects of both Cu_2_O and Cu_2_S substrates, particularly against the Gram-negative bacterial model *E. coli*.

**Figure 6:**
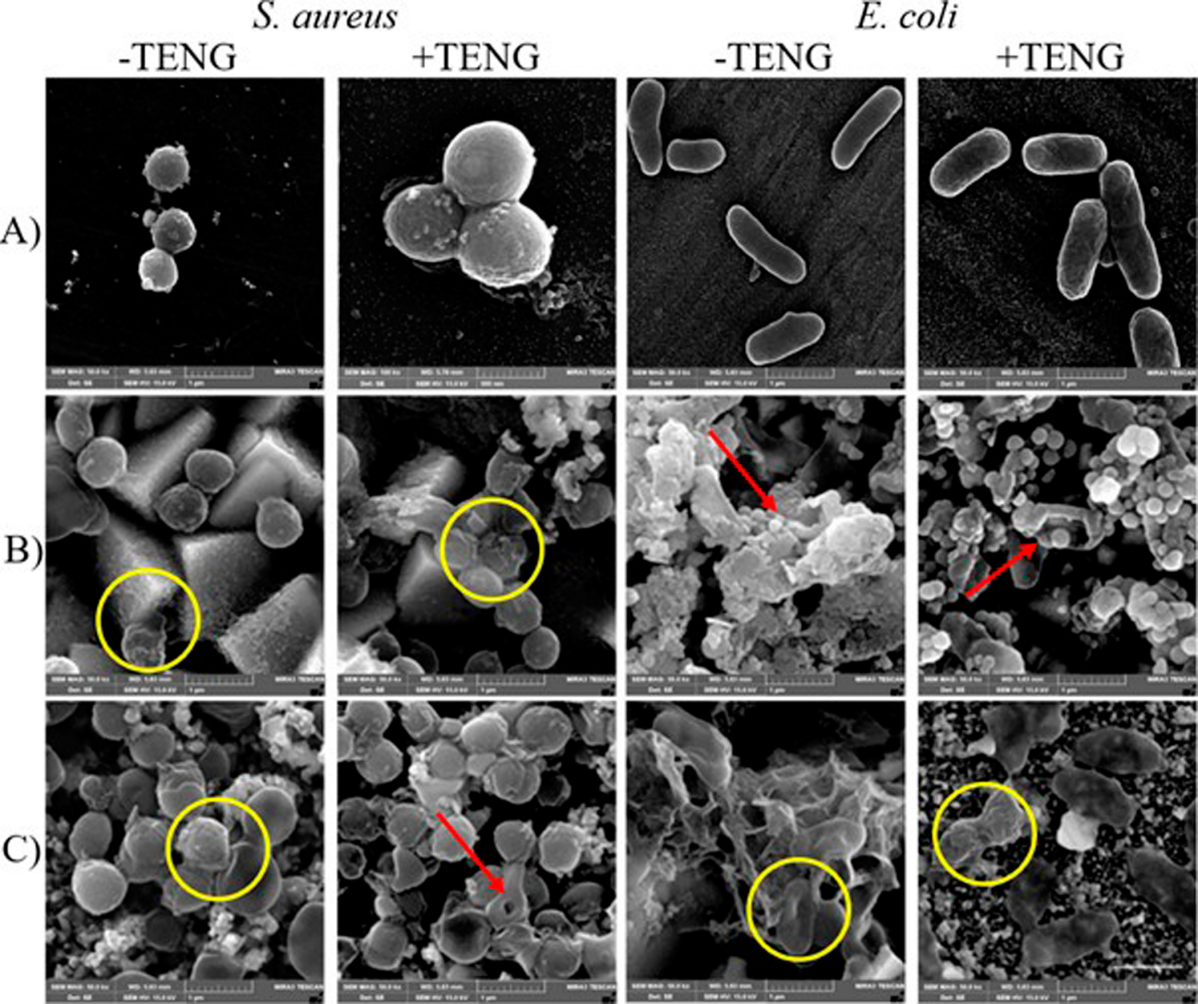
FESEM images showing the micromorphological structures of incubated *E. coli* and *S. aureus* bacteria on (A) FTO, (B) Cu_2_O, and (C) Cu_2_S surfaces with and without 10 min of TENG-based electrical stimulation.

### 2.6 Antibacterial Mechanism

To examine the antibacterial mechanism(s) of Cu-based substrates (Cu_2_O and Cu_2_S), the concentration of the released Cu ions was quantified both in the absence and presence of the TENG-based electrical effect. Inductively coupled plasma (ICP) analysis was employed to quantify ion concentrations, enabling a comparative assessment of ion release under varying conditions his analysis provided critical insights into how the TENG effect influences and enhances antibacterial activity through ion modulation.

As previously mentioned, the antibacterial activity of Cu-based host electrodes against both bacterial models was enhanced by inducing a triboelectric effect, which generated an electrical response in the electrode. This enhancement in antibacterial efficacy was particularly evident in Cu_2_S electrodes subjected to electrical stimulation.

ICP analysis revealed that the release of Cu ions from the Cu_2_S electrode increased substantially when a TENG-based electrical current was applied, rising from 1.3 to 2.8 μg/mL (∼1.15 times) after 20 min of triboelectric stimulation. In contrast, copper ion release from the Cu_2_O electrode remained relatively unchanged following the triboelectric effect, increasing only from 8.2 to 9.6 μg/mL (∼0.17). This suggests that Cu_2_O releases a large amount of Cu ions even without electrical stimulation, probably due to its lower stability in aqueous environments.^39^ Meanwhile, the more stable Cu_2_S electrode^39^ only released significantly higher amounts of Cu ions when activated by the triboelectric effect.

The reciprocating motion of Kapton on Cu_2_O or Cu_2_S layers generates an alternating current, reflecting the back-and-forth movement of electrons during each tapping cycle to maintain charge balance in the TENG setup. During the release phase of each tapping cycle, primary electrical stimulation occurs, generating an associated electric field, and resulting in distinct current spikes. These spikes indicate that the primary electrostatic charges on the host electrodes are positive. Indeed, in the case of nonequilibrium molecular-dynamics simulation with applied electric field in both directions across the interfacial FTO–Cu_2_O system to induce polarization thereat, the Hirshfeld charges^40^ were found after 1 ps of relaxation; the field-induced change in atomic charge was ∼0.048 ± 0.026 e in magnitude. This provides for substantial charge-transport effects as a result of triboelectric-field action. In view of this field-induced charge transfer, the adherence of negatively charged bacteria (i.e., *E. coli* and *S. aureus*)^41^ to the positively charged host surfaces can be significantly enhanced through electrostatic attraction.^16,42^ Once the bacteria adhere to the surface, their proximity to the Cu ions (Cu^+^ and Cu^2+^) released from the surfaces increases, allowing for more direct interaction with these ions. Cu_2_S, with its narrower band gap (∼1.2–1.5 eV) and higher electronic conductivity compared to Cu_2_O (∼2.0–2.2 eV),^43–45^ facilitates more efficient charge-carrier transport under electric stimulation, such as that induced by the TENG. Due to the enhanced conductivity,^46^ positively charged Cu_2_S surfaces (as a result of the triboelectric effect) can exhibit stronger electrostatic adhesion to the negatively charged bacterial membranes. This intimate interaction enhances the localized concentration of copper ions (Cu^+^ and Cu^2+^) at the bacterial interface, facilitating their uptake and interaction with cellular components, ultimately leading to membrane disruption, protein inactivation,^47^ and bacterial death (Figure 6).

Meanwhile, the TENG-based electrical stimulation can interfere with electron-transport chain (ETC) in bacteria. Upon receiving the TENG-based electrical stimulation, positively charged electrode surfaces can trap electrons from the bacterial ETC, disrupting the normal flow of the electrons, involved in bacterial respiration.^48,49^ This interference with the respiratory process compromises the bacteria’s energy production, ultimately impairing their metabolic supply chain.^50,51^ Additionally, trapped electrons on the surface can be shuttled toward available molecular oxygen, leading to ROS generation, further intensifying oxidative stress, and contributing to bacterial death.^52,53^ Induced oxidative stress, a key pathway commonly associated with antibacterial activity,^54^ was also investigated through qualitative and quantitative analysis to indicate the generation of ROS under various treatments. Intracellular ROS were qualitatively assessed by detecting green fluorescence, which is emitted when the nonfluorescent dichlorofluorescein diacetate (DCFH-DA) is oxidized upon interacting with ROS produced inside bacterial cells^55^ (cf. Figure 7). Therefore, the intensity of green fluorescence quantitatively indicates the level of oxidative stress within the bacterial cells. On the other hand, quantitative studies based on employing two specified molecular probes, i.e., terephthalic acid (TPA) and 1,3-diphenylisobenzofuran (DPBF) shown in Figure S3, revealed hydroxyl radicals (^•^OH) and singlet oxygen (^1^O_2_), respectively.^52^ The results showed a strong qualitative and quantitative (Figures 7 and S3) correlation with the observed antibacterial effects across various treatments. This shows that bacterial cells experienced higher oxidative stress when exposed to Cu_2_O, compared to the Cu_2_S substrate, independent of the triboelectric effect. However, when the TENG’s electric impact was applied to the Cu_2_S substrate, the intensity of intracellular ROS generated in the bacterial cells significantly improved, leading to enhanced antibacterial performance of the Cu_2_S electrode. Indeed, the enhance charge transport in Cu_2_S^46^ can enhance the generation ROS due to the faster electron transfer and redox cycling processes.^56^ Therefore, these antibacterial activities can be linked directly to the combined effects of copper ion release and oxidative stress induction, both of which are triggered and boosted by applying the triboelectric effect, the resultant electric field manipulates ion-release mechanics and kinetic and the concomitant oxidative effect (and stress). Together, these synergistic effects likely account for the more pronounced antibacterial activity of Cu_2_S under TENG-induced electrical stimulation. While Cu_2_S demonstrated strong stimulation-activated antibacterial activity, its long-term application may be challenged by potential susceptibility to environmental structural changes and/or degradation induced by repeated TENG operation. These durability concerns warrant further investigation to ensure successful practical translation and consistent performance in real-world scenarios.

**Figure 7:**
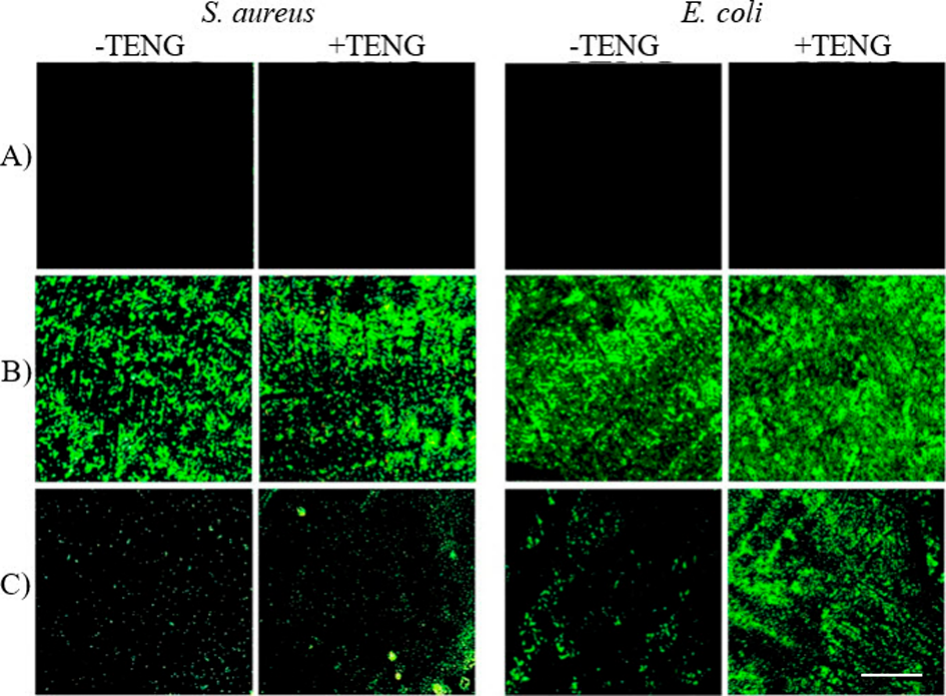
Qualitative assessments of induced ROS in bacterial cells exposed to host electrodes of (A) FTO, (B) Cu_2_O, and (C) Cu_2_S, visualized via fluorescent microscopy. The intensity of green radiation serves as a qualitative proxy measure of the ROS levels generated in the bacterial cells. The scale bar indicates 100 μm.

Reflecting further on the above (and in Figure 7), the superior antibacterial effects observed on *E. coli* when exposed to both Cu_2_O and Cu_2_S surfaces can be attributed to the distinct structural differences between Gram-negative and Gram-positive bacteria. Thicker peptidoglycan layers (20–80 nm) in Gram-positive bacteria, such as *S. aureus*,^57^ compared to the few nanometers found in Gram-negative bacteria, serve as an outermost structural component,^58^ providing increased resistance to the penetration of copper ions. This resistance effectively retards the antibacterial action in *S. aureus,* as Gram-positive, bacteria compared to that observed in *E. coli* as Gram-negative bacteria.

The results highlight the crucial role of material–electrical stimulation synergy in driving antibacterial performance. The use of FTO, Cu_2_O, and Cu_2_S substrates, each with distinct electronic and chemical properties, allowed us to examine their individual and combined effects with TENG-based electrical stimulation. FTO, being inert and nonantibacterial, served as a control and exhibited no bacterial inhibition, even under electrical stimulation. In contrast, Cu_2_O demonstrated inherent antibacterial activity that was not significantly enhanced by electrical stimulation, suggesting it may operate near its maximum efficacy under static conditions. Interestingly, Cu_2_S, which did not demonstrate strong antibacterial activity without stimulation, showed pronounced antibacterial effects when coupled with TENG-induced electrical stimulation. This response is attributed to its higher electrical conductivity and narrower bandgap,^44^ which enhances charge transport and localized electrochemical activity under stimulation, thereby facilitating Cu ion release and ROS generation. These results suggest that Cu ion cannot fully explain the observed antibacterial effects, particularly in the case of Cu_2_S, where ion release remained relatively low compared to Cu_2_O. This points to a synergistic interaction between the material properties and the applied stimulation. To better understand the individual contributions of each antibacterial mechanism such as Cu ion toxicity, ROS-mediated oxidative stress, and electrostatic effects, future studies should be considered. These could include the use of Cu-chelating agents to isolate the effects of copper ion release and ROS scavengers to assess the role of oxidative stress. Such targeted approaches would help decouple and quantify the specific contributions of each mechanism to the overall antibacterial performance. using selective inhibition strategies, involving the use of Cu-chelating agents to isolate ion effects and ROS scavengers to examine oxidative stress. These targeted approaches can be applied to decouple and quantify the specific contributions of each mechanism.

## 3 Conclusions

This research demonstrates the integration of triboelectric nanogenerators (TENGs) with Cu-based substrates (Cu_2_O and Cu_2_S) as an innovative, battery-free approach to antibacterial functionality in wearable electronics. The study successfully developed a novel sulfurization method to fabricate Cu_2_S substrates, which exhibited remarkable antibacterial efficacy under TENG-driven electrical stimulation. The enhanced antibacterial activity of Cu_2_S was attributed to a substantial increase in Cu ion release, intensified electrostatic interactions with bacterial membranes, and electron capture mechanisms that disrupted bacterial energy metabolism and induced oxidative stress. These findings highlight the feasibility of integrating TENG-enabled platforms into biomedical and wearable technologies, offering a sustainable solution with inherent antibacterial properties. This improvement addresses critical safety concerns associated with direct human contact and paves the way for developing next-generation wearable devices that combine energy harvesting with on-demand antibacterial functionality.

## 4 Materials and methods

### 4.1 Substrate Preparation

The FTO/glass substrates (TCO30-10, 10 Ω/sq) were cleaned using a sequential process involving detergent, acetone, and ethanol in an ultrasonic bath for 10 and 5 min, respectively. Finally, the substrates were rinsed with deionized (DI) water and dried under a nitrogen stream.

#### 4.1.1 Cu_2_O Layer Fabrication

The electrodeposition process was conducted using a three-electrode setup comprising a Pt mesh as the counter electrode, an Ag/AgCl electrode in 3 M KCl as the reference electrode, and the FTO/glass substrate as the working electrode. A potential of −0.5 V vs Ag/AgCl was applied to the electrochemical bath, using a potentiostat–galvanostat (Autolab-PGSTAT30). The bath solution contained 0.3 M CuSO_4_ (Aldrich) and 3 M lactic acid, with its pH adjusted to 12 by adding NaOH and sulfuric acid (Sigma-Aldrich). The deposition temperature was maintained at approximately 60–63 °C. After deposition, the layers were rinsed with DI water and dried under ambient conditions. The film morphology was investigated using FESEM analysis.

#### 4.1.2 Cu_2_S Layer Fabrication

The Cu_2_S layers were fabricated by sulfurizing an already prepared Cu_2_O layer within a quartz tube furnace under an argon atmosphere. This process employed a methodology similar to the sulfurization of FTO/glass substrates to produce SnS_2_/FTO/glass structures.^59^ To initiate the process, 500 mg of sulfur was placed as the source material, with the substrate positioned 20 cm downstream of the sulfur source. The quartz tube was purged with pure argon gas for 30 min to ensure complete removal of any residual oxygen. Sulfurization was conducted at 500 °C for 60 min, during which sulfur vapor species (S) diffused into the Cu_2_O layer, facilitating its transformation into Cu_2_S. Following the sulfurization process, the sample was allowed to cool naturally to room temperature. A schematic representation of the Cu_2_S fabrication process is provided in Figure S4. The sulfurization temperature of 500 °C was selected based on established protocols for synthesizing similar metal sulfides, such as SnS_2_, via a chemical vapor deposition method.^60^ This temperature allows for effective sulfur diffusion and complete phase transformation while minimizing secondary phase formation. Moreover, previous studies have demonstrated that FTO-coated glass substrates remain structurally and electrically stable at temperatures up to 550 ± 50 °C.^61,62^ The preserved conductivity of the FTO layer after sulfurization confirms its integrity under the applied conditions.

### 4.2 Computational Modeling

Density functional theory (DFT) was carried out using the general gradient approximation with the Perdew–Burke–Ernzerhof functional.^63^ This DFT was performed in a nonequilibrium fashion in applied electric fields to mimic the triboelectric effect at play in TENG, in the sense that we wish to account for the change in point charges; here, the field was applied in both directions (perpendicular to the interface) to mimic experimental negative- and positive-charging. This was accomplished by using coordinates from the geometry-preparation procedure for FTO as described in ref 64 with supercell replication, while two Cu_2_O layers were deposited thereon in two layers in the *Pn*3*m* space group with an interfacial strain less than ∼2%. *CP2K* software was used with second-generation Car–Parrinello dynamics in the presence of external electric fields using the Berry-phase method for electric-field implementation.^65^ The time step was 1 fs and the field intensity was 0.08 V/Å, which results in nuclear forces no more than about 2.5–4% of those arising from intrinsic electric fields and is in the linear-response regime, albeit on the cusp in the transition toward a nonlinear response if higher applied-field intensities were to be used,^65^ given the need to observe an appreciable “signal-to-noise” ratio in the charge polarization due to the shifting (tribo)­electric-field phenomena.

### 4.3 Integrated TENG Coupled by a Antibacterial Host

A Kapton film was wrapped snugly around a human finger to serve as the single-electrode, triboelectric active layer. This Kapton-coated finger was manually tapped against a grounded FTO glass substrate at ∼4 Hz. Upon each contact, electrons transferred from the FTO (more tribopositive) to the Kapton (more tribonegative), and upon separation the negatively charged Kapton drew electrons from ground back into the FTO, completing the circuit and generating an alternating current in the external circuit. Concurrently, Cu_2_O or Cu_2_S microstructure films were deposited onto separate FTO/glass substrates, which acted as “host electrodes”. Each host electrode was electrically connected to the TENG’s FTO electrode via an external circuit, allowing the alternating current produced by the Kapton–FTO contact-separation cycle to be delivered directly to the Cu-based films. The resulting current and voltage outputs were thus applied to the Cu_2_O or Cu_2_S host electrodes, enabling evaluation of their antibacterial performance under TENG-induced electrical stimulation.

### 4.4 Material Characterization

X-ray diffraction (XRD; X’Pert PRO, PANalytical), local optical microscopy, and field-emission scanning electron microscopy (FESEM; TESCAN, MIRA3) were utilized to investigate the crystallization, and structural characteristics (including morphology), of the Cu_2_O and Cu_2_S layers, which serve as the host electrodes. These techniques allowed for a comprehensive analysis of the material properties and surface features of the layers. Additionally, a potentiostatic–galvanostatic system (μAutolab system, Metrohm) was employed to accurately measure the current within the designed circuit, providing valuable insights into the electrical performance of the electrodes.

### 4.5 Antibacterial Activity Investigation

The antibacterial efficacy of the host electrodes under TENG-based electrical stimulation was thoroughly evaluated, contrasting with those electrodes that did not receive any electrical stimulation. Our investigation employed two principal methodologies—optical density (OD6_00_) measurements and CFU counting—conducted on *Escherichia coli* (ATCC 25922) and *Staphylococcus aureus* (ATCC 25923) bacteria, representing Gram-positive and Gram-negative strains, respectively.

Prior to commencing the antibacterial investigations, thorough sterilization procedures were carried out. This involved autoclaving all glassware, wires and culture media, including phosphate-buffered saline (PBS, Merck), Muller–Hinton broth (MHB, Merck), and agar for 20 min. Additionally, to avoid any unforeseen alterations in the chemical structures of the sample electrodes, both sides of the electrodes underwent sterilization by exposure to a UV lamp for 30 min.

The initial step of the antibacterial test involved preparing a suspension of bacterial models at their midphase log with a concentration of 0.5 × 10^8^ CFU. This concentration was chosen to simulate a high bacterial load, which is commonly used to evaluate the antibacterial performance of materials under challenging conditions. While the natural bacterial load on healthy skin typically ranges from 10^2^ to 10^6^ CFU/cm^2^,^66^ it is important to note that even low initial concentrations of pathogenic bacteria can rapidly proliferate and become harmful under favorable conditions such as warmth, moisture, and the presence of nutrients. Many pathogens, including *E. coli* and *S. aureus*, can multiply exponentially, often doubling in number every 20–30 min under optimal conditions.^67,68^ Therefore, employing a high initial concentration in this study reflects potential real-world infection scenarios (e.g., contaminated wounds or medical device surfaces). Moreover, it ensures that the antibacterial efficacy of the material is assessed under clinically relevant and rigorous conditions, thereby enhancing the reliability and significance of the results.

Subsequently, the surface of the host electrode, linked to the TENG electrodes via a sterilized wire, was inoculated with 100 μL of this suspension by carefully spreading it. After incubating the bacteria, a Kapton-wrapped finger tapping was applied to the FTO electrode, which served as the TENG electrode, in order to generate an electrical current. The instantaneous transfer of the generated electrical stimulation from the TENG electrode to the bacteria incubated on the surface of the host electrodes was facilitated by the connecting wire. Following 10 min exposure to electrical stimulation, the bacteria were delicately washed off the surfaces of the host electrodes using PBS (a pH of ∼7.4) into a sterilized Petri dish.

Subsequently, 10 μL of the washed (bacterial) suspension was added to the 100 μL of MHB already present in specific wells of a 96-well plate, facilitating the OD_600_ measurement. Concurrently, another 10 μL of the suspensions were gently and evenly spread across the solid agar surfaces in the plates to determine the colony-forming units of the bacteria. To further distinguish between the antibacterial effects resulting from TENG stimulation and those inherent to the host materials, following exposure to electrical stimulation, the bacteria were maintained in contact with the host electrodes for an extended period (overnight) without additional triboelectric effect. This was achieved by pouring molten agar (∼43 °C) over the host electrodes (which had already been inoculated with bacteria and subjected to treatments) inside sterilized Petri dishes, allowing the agar to solidify. Subsequently, all samples were transferred to an incubator set at 37 °C and stored overnight.

To gain deeper insights into the antibacterial characteristics of the host electrode in response to electrical stimulation, fluorescent staining was employed to distinguish between dead/damaged and live bacteria. Bacteria cultured on the host electrodes, regardless of exposure to electrical stimulation treatment, were harvested by centrifugation at ∼5 °C and 7000 rpm for 20 min to form a bacterial plate in the sediment. This bacterial plate was then resuspended in PBS and subjected to staining with AO/PI as fluorescent dye indicators at 37 °C for 10 min. Subsequently, unbound fluorescent dyes were removed by centrifugation, leaving the dye-bound (stained) bacteria collected in the sediment, which were then resuspended in PBS. Next, 10 μL of the resultant suspension was evenly distributed on an already clean glass slide and visualized under a fluorescence microscope (Zeiss, Germany) equipped with a charge-coupled-device (CCD) camera. Exhibiting live bacteria emitted green fluorescence, whereas dead/damaged bacteria emitted red fluorescence.

### 4.6 Microstructure Studies of the Bacteria

To deepen our understanding of the antibacterial effects induced by electric stimulation, an investigation was conducted into the microstructures of bacteria when exposed to the host electrodes, both with and without electrical stimulation. Following the incubation of bacteria on the host electrodes using the identical conditions employed for antibacterial testing, the bacteria were rinsed off the electrodes with 1 mL of PBS and then centrifuged at ∼5 °C and 500 rpm. After discarding the supernatant, the bacterial plates in the sediment were resuspended carefully in PBS. Subsequently, the resultant suspensions were evenly distributed onto (already clean in an ultrasonic bath using detergent followed by rinsing with DI water and ethanol) glass slides. Afterward, the bacteria spread on the glass slide surfaces were immobilized for about 4.5 h using a solution of 2.5% glutaraldehyde [a mixture of glutaraldehyde (25%) with PBS and DI water in a ratio of 1:4:5]. The fixation was finalized by a series of dehydration processes using sequential aqueous ethanol concentrations of 30%, 50%, 70%, 90%, and ultimately 100%. The prepared samples were then coated with gold for investigation using a FESEM MIRA3 (Brno, Czech Republic).

### 4.7 In Vitro-Induced ROS Study

The generation of ROS in bacterial models (due to the finger-tapping electrical stimulation) was in vitro investigated through two primary methods including quantitative and qualitative. In the former method, the presence of induced ROS within bacterial cells was visualized using the fluorescent dye DCFH-DA, which reacts with the generated ROS, emitting a vibrant fluorescent green color indicative of its oxidized form [2′,7′-dichlorofluorescein (DCF)].^52^ Following exposure to the host electrodes, with or without electrical stimulation, the bacteria were washed off the surface, centrifuged (at approximately 5 °C and 800 rpm), and then treated with DCFH-DA dye (10 × 10^–6^ M) for 15 min after resuspension. Toward the end, the level of emitted green fluorescence was observed by exciting the samples at the wavelength of 488 nm using a fluorescent microscope (Zeiss, Germany) equipped with a CCD camera. Toward the end, the level of emitted green fluorescence was observed by exciting the samples at the wavelength of 488 nm using a fluorescent microscope (Zeiss, Germany) equipped with a CCD camera.

To quantitatively assess specific types of ROS within the bacterial cells, namely, ^1^O_2_ and ^•^OH, two distinct molecular probes were employed: DPBF and TA, respectively. Upon interaction with the induced ^1^O_2_ and ^•^OH, the strong fluorescent emission of the molecular probes of DPBF at ∼490 nm drops. Also, the fluorescent peak of nonfluorescent TA emerged and also increased at a wavelength of 448 nm, indicating the presence and concentration of the respective species.

### 4.8 Statistical Analysis

The statistical analysis of the data was conducted using the *SPSS* software package (version 26; SPSS, Inc., Chicago, IL, USA). To ascertain significant statistical effects, one-way analysis of variance (ANOVA) was employed based on 99% and 95% (*p* < 0.01 and *p* < 0.05, respectively), confidence levels. The reported data were presented as mean ± standard deviation (SD), derived from a minimum of three tests. All antimicrobial experiments, including colony counting, were performed in triplicate (*n* = 3) under each condition, and data are reported as mean ± SD.

## Supplementary Material



## Notes

Additional SEM images of Cu_2_S samples, a single voltage pulse generated at ∼4 Hz, quantitative comparison curves of ROS generated in bacterial cells with and without TENG, and schematic representation of a Cu_2_S layer growth on a FTO/glass substrate (PDF)
