# Supplementary material for: Enhanced On-Demand Antibacterial Platform Based on Triboelectric-Nanogenerator-Induced Electrical Stimulation of Cu2S Substrates
Source: ACS Appl Mater Interfaces. 2025 Jul 21;17(30):42624–36. doi: 10.1021/acsami.5c04717 (PMC12314864; doi:10.1021/acsami.5c04717)
Supplement: Supplementary file 1 [file am5c04717_si_001.pdf]

Supporting information

Enhanced On-Demand Antibacterial Platform Based on Triboelectric Nanogenerator-Induced Electrical Stimulation of Cu_2S Substrates

Marziyeh Jannesari [a, b]†*, Leyla Shooshtari [b, c]†, Nima Mohamadbeigi [b], Niall J. English [a], Raheleh Mohammadpour [b],

[a] School of Chemical and Bioprocess Engineering, University College Dublin, Belfield, D04 V1W8 Dublin, Ireland

[b] Center for Nanoscience and Nanotechnology, Institute for Convergence Science & Technology, Sharif University of Technology, 14588- 89694, Tehran, Iran

[c] Semiconductor department, Materials and Energy Research Center, 31787-316, Karaj, Iran

† These authors contributed equally to this work.

* Corresponding author:

E-mail address: marziyeh.jannesari@ucd.ie , mjannesari2000@gmail.com (orcid.org/0000-0002-7998-2961)

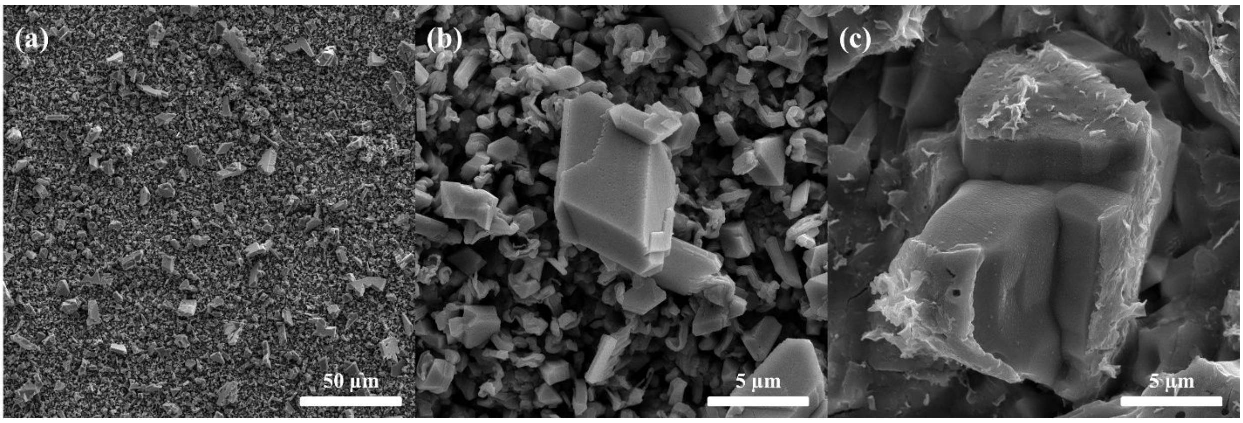

Figure S1: Top view FESEM images of Cu_2S at different magnifications (**a**) 1 kx, (**b, c**) 10 kx.

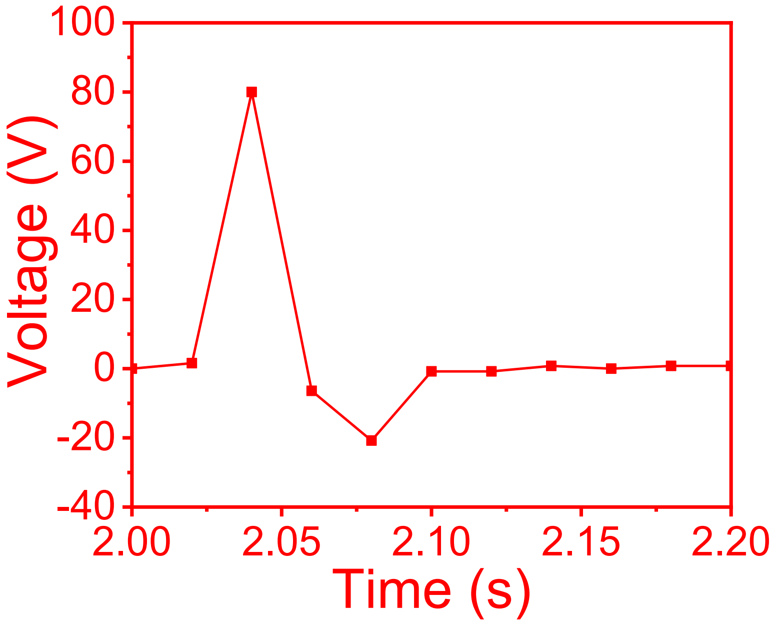

Figure S2. Evolution of a single voltage pulse generated by tapping a Kapton-wrapped finger on the FTO substrate at a frequency of ~ 4 Hz. https://doi.org/10.2174/97897815040739122010014

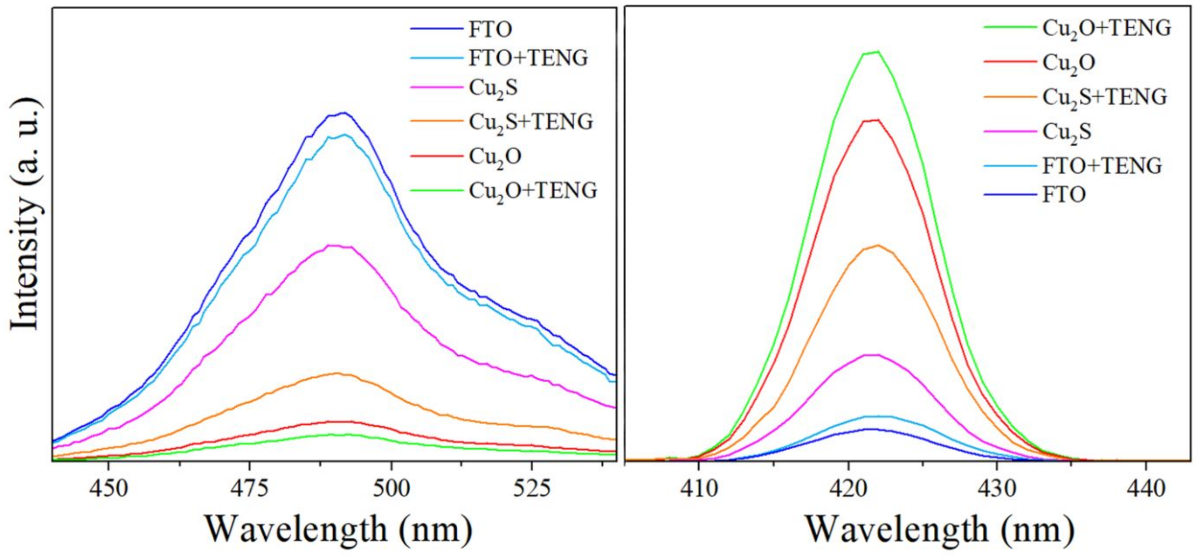

Figure S3. The quantitative measurements of hydroxyl radicals ($^{\cdot}OH$) and singlet oxygen ($^1O_2$) levels generated in bacterial cells exposed to host electrodes of FTO, Cu_2O, and Cu_2S were conducted using terephthalic acid (TPA) and 1,3-diphenylisobenzofuran (DPBF) molecular probes for E. coli, respectively. The scale bar represents 100 μm.

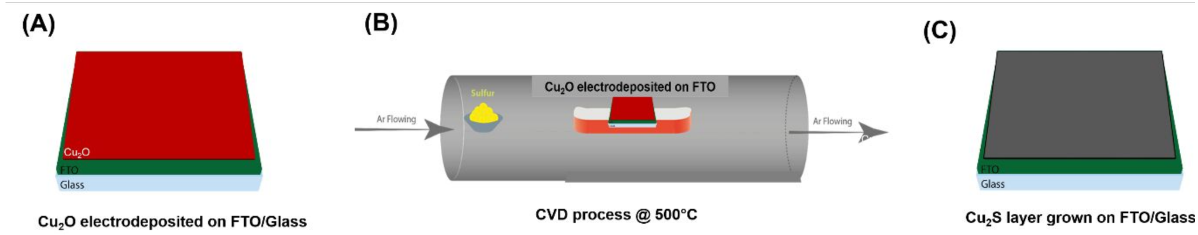

Figure S4. The schematic representation of the Cu_2S layer growth on the FTO/glass substrate, (A) the fabrication of Cu_2O film via electrodeposition process, (B) sulfurizing of the Cu_2O/FTO/glass layer, and (C) the resulting Cu_2S layer on FTO/glass achieved through the sulfurizing process.
